# Political crisis and the Corona—‘State of Emergency’ in Kosovo

**DOI:** 10.1007/s42597-020-00046-w

**Published:** 2020-10-30

**Authors:** Werner Distler

**Affiliations:** Center for Conflict Studies, Philipps-Universität Marburg, Ketzerbach 11, 35032 Marburg, Germany

**Keywords:** Kosovo, Corona, State of Emergency, Kosovo, Corona, Ausnahmezustand

## Abstract

Amidst the arrival of the Coronavirus (SARS-CoV-2) in Kosovo, the new coalition government of Vetevendosje and the Democratic League of Kosovo (LDK) under the leadership of Prime Minister Kurti (Vetevendosje) collapsed after a vote of no confidence on 25 March 2020. On the surface, the vote, initiated by LDK, was the consequence of a conflict over the appropriate Corona strategy. A two-month power struggle under the extraordinary circumstances of Corona-related restriction followed, resulting in a new LDK-led government in early June. The political crisis in Kosovo in the early-phase of Corona has to be analyzed against the background of a political crisis with legacies in the post-conflict period, the polarization between Vetevendosje and other parties, and the strong influence of external actors in Kosovo. In this forum article, I ask how the discourse on the “state of emergency” and the pandemic has influenced power struggles in Kosovo and how the conflicts have challenged the democratic competition and institutions. My main argument is that the pandemic indeed did not lead to any form of cooperation, but the political competitors used the pandemic for an intensification of their power struggle, which—for now—led to the restoration of a government of established elites, supported by diplomatic interventions from external actors.

## Abstract (de)

Ende März 2020, kurz nachdem die ersten Infektionsfälle mit dem Corona-Virus (SARS-CoV-2) im Kosovo auftraten, wurde die gerade erst ins Amt gekommene Regierung unter Führung von Premierminister Kurti (Vetevendosje) durch ein Misstrauensvotum des Koalitionspartners von der Democratic League of Kosovo (LDK) zu Fall gebracht. Auf den ersten Blick war dies die Folge einer Auseinandersetzung über eine angemessene Corona-Strategie. Ein zweimonatiger Machtkampf folgte, der schließlich zu einer neuen LDK-geführten Regierung im Juni führte. In diesem Artikel zeige ich, dass die politische Krise in einer so kritischen Phase vor allem vor dem Hintergrund lang bestehender politischer Rivalitäten und dem übermäßigen Einfluss externer Akteure verstanden werden muss. Ich frage, wie der Diskurs über den „Ausnahmezustand“ im Kontext der Pandemie den Machtkampf im Kosovo beeinflusst und demokratische Institutionen und Prozeduren herausgefordert hat. Dabei zeigt sich, dass die politischen Akteure im Kosovo die Pandemie nicht für eine – selbst vorübergehende – Kooperation, sondern für eine Eskalation der Rivalität genutzt haben, die einen „restaurativen“ Effekt hatte. Für den Moment haben die etablierten Eliten die Kontrolle zurückgewonnen, unterstützt durch externe Akteure.

## Introduction

Kosovo has experienced fundamental societal and political transformations over the last three decades. From violent conflict in the 1990s, to the interventions and direct international governing in the 2000s, to independence^1^ and, in the last decade, to a consolidating period of rising domestic power—however, still under “diplomatic interventionism” (Visoka 2017a, p. 8) by other states and International Organisations and pressure of successful Europeanisation (Baracani 2020). Domestic politics are dynamic and highly competitive, with short-term governments and early re-elections, and conflicts centering on accusations of “state capture” by political elites, especially those who have emerged from the former Kosovo Liberation Army (KLA) (Tadić and Elbasani 2018). Against this background, I want to re-construct how the Corona (SARS-CoV-2) pandemic influenced the competition between political opponents, which even led to the downfall of a government in March, and how the power struggle challenged the state institutions.

In March, the pandemic reached Kosovo at a time of a new political dawn. In early February 2020, a new government of the long-time opposition party Vetevendosje (Self-Determination), winner of national elections in October 2019, and the Lidhja Demokratike e Kosovës (LDK, Democratic League of Kosovo) had been sworn in. This government was exceptional in several ways. For the first time since the independence of Kosovo in February 2008, none of the parties that had emerged from the Kosovo Liberation Army (KLA) was part of the government. Furthermore, it was the first coalition of Vetevendosje and LDK and the first participation of Vetevendosje overall. However, the new coalition government only lasted a few weeks: On March 25, the LDK initiated a successful vote of no-confidence in the parliament. The vote followed the decision of Prime Minister Albin Kurti (Vetevendosje) to dismiss Interior Minister Agim Veliu (LDK) after he supported calls by President Thaci (Partia Demokratike e Kosovës, PDK, Democratic Party) for a “state of emergency” to combat Corona. With the LDK leaving the government, Kosovo entered a period of caretaker government under Vetevendosje. In early June 2020, a new coalition led by the LDK took power without new elections, but sanctioned by the Constitutional Court.

The guiding questions of this forum article are: How has the discourse on the “state of emergency” due to Corona influenced existing political conflicts and how has the power struggle challenged the democratic competition and institutions since the entry of Corona in Kosovo? Taking Kosovo as a case of post-conflict state in a period of consolidation, my main argument is that in the context of domestic political competition, the pandemic did have a conflict-intensifying and “restorative” effect. Political actors could not agree to seize conflicts and power struggle, even for a brief period, but instead established elites used the pandemic to oust Vetevendosje from the government, with partial support from external actors. While the change in government was realised within the constitutional order, the legitimacy of institutions in Kosovo may have suffered in consequence. The development of Corona infections or anti-Corona policies in Kosovo are not the focus of this article. However, it should be noted that amidst the power struggle, the numbers of infected and of deaths grew enormously until summer 2020 and the pandemic is not yet under control in Kosovo and neighboring states.^2^

First, I will give a short overview on what we could expect regarding the influence of a pandemic on competitive domestic politics. In the main part, I will provide some background information on the political opponents and then reconstruct the power struggle from March until June 2020.

## How do epidemics/pandemics influence domestic politics and power struggles?

With global numbers of Corona infections on the rise in September 2020 and no end to the pandemic in sight, it is too early to reflect upon mid- and long-term implications for domestic and international politics and conflicts. Even the short-term consequences of Corona on violent conflicts seem to be mixed, with cases of reduced, but other cases of increased violence (Ide 2020; Basedau and Deitch 2020). Due to the extraordinary character of the pandemic, research cannot simply draw on existing studies. However, we find important insights in studies on previous epidemics/pandemics and disasters. Here, I will focus primarily on the question of how pandemics/epidemics influence domestic politics, not on how government’s reactions influenced the development of pandemics/epidemics.^3^ If we understand the rise of Corona in February/March as an extraordinary crisis, then interdisciplinary literature gives us examples on how political opponents can engage in short-term cooperation and mutual support (for example of governments by oppositions) in such periods of emergency in democracies, e.g. in the United States after the attacks on 11 September 2001 (Entman 2003; Owens 2009) or in the economic crisis after 2009 in European states (Moury and De Giorgi 2015). These phenomena could be defined as “antagonistic cooperation” between political adversaries to pursue common interests for a specific time in a clear institutional set-up (Best 2009, p. 425).

Some studies on politics under the influence of pandemics/epidemics highlight that governments tend to react robustly, especially when under pressure from international and domestic actors. In 2006, when faced with the H5N1 avian influenza virus, the Indonesian government chose to rather aggressively “nationalize” and re-centralize its health policies and funding, ignoring international and domestic critique, to emphasize “sovereign” control (Hameiri 2014). Another example comes from the cholera outbreak in Zimbabwe in 2008: Instead of short-term cooperation, the main opposition party, the Movement for Democratic Change under Morgan Tsvangirai (MDC-T) and the ruling party, Zimbabwe African National Union—Patriotic Front (ZANU-PF) politicized cholera heavily and integrated narratives of mismanagement of the epidemic in their discourse to discredit opponents (Masakure 2018). Moran shows, how much the Ebola epidemic resulted in a lack of trust or even mistrust in the government of Sierra Leone in the early period of Ebola in 2014 (Moran 2015), a further hint to potential consequences for domestic power struggles.

To summarize, while there is a possibility for short-term “antagonistic cooperation” between political opponents in a democratic set-up when faced with an extraordinary crisis, studies on epidemics/pandemics do also highlight rising mistrust, strong state-centered reactions, and the dominance of political grievances over cooperation.

## The political crisis in Kosovo during the early-phase of the Pandemic

### Background

The elections in October 2019, with the winning party Vetevendosje (relative majority of 25.8%), opened up the possibility for a new government coalition in Kosovo, led by the long-term opposition party. In general, forming governments in Kosovo has often been a challenge; not least because of the 20 reserved seats (out of 120) for national minorities in the parliament and a dominating Kosovo-Serbian party with close ties to Belgrade. The LDK and parties that emerged from the KLA, like the PDK or the AAK, were bitter rivals after the war and throughout the 2000s. During this period, the LDK lost its formerly dominant position in the political system to the PDK, who dominated governments since 2007. The parallel rise of Vetevendosje to a successful political force in Kosovo has frustrated many domestic and international actors. Since its creation in 2005, the movement organized protests against intervening actors, especially against the United Nations Mission in Kosovo (UNMIK), and later against the European Union mission EULEX. While the movement is characterized as an important “counteracting” agent of resistance against liberal peacebuilding and seemingly never-ending interventionism (Björkdahl and Gusic 2015), its rhetoric and uncompromising activism concerned observers and competitors alike (Distler and Riese 2013; Visoka 2017b). However, neither aggressive rhetoric against Vetevendosje, nor the imprisonment of leading Vetevendosje politicians were able to prevent the growing success of the party and its “anti-establishment politics” (Yabanci 2016), which is also due to support from young voters in Kosovo. A growing part of the citizenry shares the critique of the “state capture”, corruption, and overall slow political and economic progress in the country, as represented by the established parties (Coelho 2018)—and the failure to achieve any breakthrough in the dialogue with Serbia over recognition. One particularly revealing example of the EU’s inability to shape state relations in its direct neighborhood is the (rocky) EU-facilitated dialogue between Belgrade and Pristina, which started in 2011, had reached an agreement in 2013 on the “Normalization of Relations” between both countries, but entered difficult terrain afterwards (Gashi and Noaković 2017). From November 2018 to July 2020, the dialogue was frozen altogether.

### Escalation

Coalition negotiations between Vetevendosje and LDK, the sole partner big enough for a new government without former government parties, proved very difficult after the elections in October 2019, partly because of conflicts over high-ranking political positions. It took until early February 2020 to form a government, only at the last moment did both parties agree on a joint program (Bami 2020).

On March 13, the first two cases of Corona were confirmed in Kosovo. Soon afterwards, the political actors in Kosovo entered a confrontational debate over the political handling of the pandemic. President Thaci (PDK) proposed a “state of emergency” in accordance with the constitution on March 17, which would have required the approval of the government and a two-third majority in parliament. Vetevendosje rejected these plans as extreme and unnecessary for a successful fight against the virus—as did the Serbian List, out of concern about an occupation of Serbian municipalities by the Kosovar army (Prishtina Insight 2020a). However, the Interior Minister Veliu (LDK) announced his support of a “state of emergency” in a TV interview, which led to his dismissal by Prime Minister Kurti (Vetevendosje) on March 18.^4^ While Veliu was known for his critical stance towards Vetevendosje, his sacking—apparently without notice to the LDK leadership—weakened the foundations of the government (Prishtina Insight 2020b). Without declaring a “state of emergency”, the government introduced further health protection measures, e.g. restrictions on traffic and movement. As a reaction, the president escalated the conflict in calling for civil and public disobedience, again demanding the declaration of the “state of emergency” (Robinson 2020a). Diplomatic interventions accompanied the crisis, with the US quite openly supporting an announced vote of no confidence against the prime minister by LDK, and the EU making more neutral calls for stability: “This is not the time for political or institutional antagonisms, this is the time for political unity.”^5^ In his speech to the parliament on March 25, Prime Minister Kurti (Vetevendosje) referred to the true reasons for the expected downfall of the government from his perspective:

To my strong belief, the engine of this rush to dismiss the government has nothing to do with the minister [Veliu, W.D.] (…), rather it is nothing less than a ready-made agreement for territorial exchange between Kosovo and Serbia. (…) Therefore, the engine behind all this deal is how to dismiss this government, how to remove me, in order to pave the way for the announcement of a state of emergency, but also for the ready-made bargain for territorial exchange. (…) With me as Prime Minister (…) no state of emergency can be announced without actually being a state of emergency, and there is no agreement in the White House (…). Even his tendency to announce the state of emergency a week ago (…) had nothing to do with the Coronavirus.^6^

Kurti was referring to one particularly problematic suggestion by Thaci and other actors to achieve an agreement with Serbia, the so-called “land swap” between Serbia and Kosovo concerning areas with Serbian and Albanian majority populations in the two countries. Recently, media claimed that there has been a Kosovar lobby campaign for this idea in other states since 2018 (Xharra 2020). Although it is not officially on the table for negotiations, and is bitterly opposed by many citizens and political actors in Kosovo, the discourse on the “land swap” shaped the political crisis in 2020, partly because of the Trump administration’s support of the idea (Joseph 2020). However, US diplomats reacted publically to the speech of Kurti on March 26: “We want to make clear there is no secret plan for land swaps between Kosovo and Serbia”.^7^

### Institutional struggle

Existing grievances and power struggles made any form of “antagonistic cooperation” between the new government party Vetevendosje and established parties in the Corona pandemic impossible. On March 25, the government lost its majority and the LDK left government positions. Kurti (Vetevendosje) then led a caretaker government. In the following, I want to reconstruct how the political opponents put pressure on the still relatively young institutions in Kosovo in their power struggle. Grievances in the political conflict mainly focused on the question of how to achieve a new government with a majority in parliament—under the conditions of Corona-related restrictions on public gatherings. Without a compromise in this question, a key role fell to the Constitutional Court. President Thaci had already petitioned the Court in the context of Corona, arguing for a “state of emergency” and against existing government restrictions—which led the Court to decide that a state of emergency was not necessary, but that a new law had to be passed so that the restrictions did not violate the Constitution (31 March).^8^ In the weeks following the no confidence vote, President Thaci (supported by some political parties) pressed for the formation of a new government and a coalition with a majority in parliament, *without *new elections. Vetevendosje, in contrast, repeatedly argued for the necessity of new elections as soon as the pandemic would allow it: “I know we are in an intensive battle against coronavirus and that can potentially delay the date of elections, but the Kosovo Constitution does not offer any other alternatives” (Robinson 2020). Prime Minister Kurti (Vetevendosje) stressed that his fight against state capture and his resistance against land swaps were the real reasons for the attempts by others to prevent new elections, which could result in even greater numbers for Vetevendosje (Prishtina Insight 2020). The active political role of President Thaci, who started talks with political parties to form a new government, was rejected by Kurti in interviews and public letters.^9^ On April 22, Thaci announced that he would give the mandate to form a government to whoever could find a majority in parliament. The LDK, having reached an agreement with the AAK (Aleanca për Ardhmërinë e Kosovës, Alliance for the Future of Kosovo), announced soon afterwards that Avdullah Hoti (LDK) planned to form such a new government. Vetevendosje sent the subsequent decree issued by Thaci, in which Hoti was proposed as prime minister, to the Constitutional Court for review. In a decision on May 1, the court suspended the implementation of the decree until May 29, in order to allow time for a comprehensive decision on the formation of a new government.^10^ In the following weeks, the parties involved exerted considerable pressure on the Court, which responded in a remarkable statement on May 19:

The Constitutional Court is following with concern the threatening public discourse (…) by the President and the caretaker Prime Minister (…) who are trying to influence or prejudice the decision-making of the Constitutional Court (…). The Constitutional Court strongly condemns this interference in its institutional independence (…).^11^

When the Court decided that the government could be formed *without* new elections on May 28,^12^ the decision and the court itself were heavily criticized by Vetevendosje as being politically influenced. On June 3, Hoti (LDK) was elected prime minister of a multi-party coalition and re-establishing the power of old elites in Kosovo. Vetevendosje has referred to the new government as “unlawful”, “unconstitutional” and “illegitimate” since^13^ and has made the LDK-led government directly responsible for the massive surge in Corona infections since June.

## Conclusion

Tragically, like other countries in the region, Kosovo has been experiencing a sharp increase in Corona infections after June. With the new government struggling to implement health policies and growing pressure from Vetevendosje, a change of government in the near future is possible—new elections could bring even better results for the opposition party. The power struggle in Kosovo is continuing, especially with the spectacular indictment of President Thaci and others for alleged war crimes by the Specialist Chambers in The Hague at the end of June, which could lead to his early resignation (if confirmed in Kosovo) and a gamble for the new presidency (Ristic 2020; Vllahiu 2020).

In summary, I would like to reflect on some broader arguments. I asked how the discourse on the “state of emergency” due to Corona influenced existing political conflicts and how the conflicts have challenged the democratic competition and institutions in spring 2020. The early discourse in March indeed supports theoretical arguments about the enormous power of a language of “emergency”, “exception”, and the “extraordinary” in shaping politics (Agamben 2005; Buzan et al. 1998). While we find many instances of resistance to emergency politics, in our case from Vetevendosje or the Constitutional Court, established political elites used the Corona crisis to reclaim the government. Instead of any form of antagonistic cooperation (Best 2009), we could observe a “restorative” effect of the pandemic in the case of Kosovo. This consequence of the pandemic could be well worth studying in other cases, especially those cases in which governments under pressure by political opponents during epidemics/pandemics have managed to keep their power. Despite this difference to other cases, the situation in Kosovo clearly supports arguments from literature on the politicization of a pandemic and the intensification of power struggles (Hameiri 2014; Masakure 2018)—comparable to highly competitive constellations like in the pre-election USA or Kosovo’s neighbor Montenegro. For a state in a period of consolidation, the crisis had put considerable amount of pressure on institutions, here especially the constitutional court, which might have suffered a loss of legitimacy as a broadly accepted interpreter of basic norms and rules. Finally, the Corona crisis revealed once more the influence of “diplomatic interventionism” (Visoka 2017a), however, with mixed outcome for the interveners. While the EU and some states like Germany lobbied for stability in March, the power struggle in Kosovo was considerably fueled by the US, with support of the no-confidence vote and constant pressure to find a “deal” between Serbia and Kosovo, culminating in the signing of an ‘economic normalization’—agreement in September 2020 in the White House (Jovic 2020). The influence of external interveners highlights the international entanglements and interdependencies of Kosovo and the whole region, which still dominate policies—despite the Corona pandemic.

## Footnotes

1 The declaration of independence from Serbia was a unilateral, but internationally supervised act. The recognition of Kosovo is still subject to political competition in the international arena. In 2018, Kosovo was recognized by 114 countries, according to data from the Foreign Ministry of Kosovo, http://www.mfa-ks.net/en/politika/483/njohjet-ndrkombtare-t-republiks-s-kosovs/483 (accessed 3 July 2020).

2 On 15 September 2020, there were 12,683 reported cases of infected persons (488 deceased), https://kosova.health/en/.

3 Compare for the latter literature on Chinas’ responses to HIV/Aids and SARS (Huang and Smith 2010) or West African States during Ebola (Moran 2015; Wilkinson and Fairhead 2017).

4 https://kryeministri-ks.net/en/kryeministri-kurti-emeron-znj-nita-shala-zevendesministre-ne-ministrine-e-drejtesise/.

5 https://eeas.europa.eu/delegations/kosovo/76422/eu-stands-together-kosovo-and-calls-all-institutional-leaders-work-together_en.

6 https://kryeministri-ks.net/en/speech-of-prime-minister-albin-kurti-at-the-assembly-of-the-republic-of-kosovo/.

7 https://www.state.gov/joint-statement-of-special-presidential-envoy-richard-grenell-ambassador-philip-kosnett-and-special-representative-for-the-western-balkans-matthew-palmer-on-kosovo/.

8 https://gjk-ks.org/en/decision/vleresim-i-kushtetutshmerise-se-vendimit-nr-01-15-te-qeverise-se-republikes-se-kosoves-te-23-marsit-2020/.

9 https://kryeministri-ks.net/en/prime-minister-kurtis-letter-to-the-president-the-double-standard-that-you-and-the-political-parties-are-trying-to-impose-on-the-unconstitutional-way/.

10 https://gjk-ks.org/en/decisions-from-the-review-session-held-on-1-may-2020/.

11 https://gjk-ks.org/en/reaction/.

12 https://gjk-ks.org/en/decision/vleresim-i-kushtetutshmerise-se-dekretit-te-presidentit-te-republikes-se-kosoves-nr-24-2020-te-dates-30-prill-2020/.

13 https://www.rferl.org/a/kosovo-s-ex-pm-kurti-stands-firm-on-demand-for-new-elections/30655325.html; https://prishtinainsight.com/hoti-elected-prime-minister/; https://prishtinainsight.com/vetevendosje-initiate-no-confidence-motion/.
